# Warburg-like Metabolic Reprogramming in Endometriosis: From Molecular Mechanisms to Therapeutic Approaches

**DOI:** 10.3390/ph18060813

**Published:** 2025-05-28

**Authors:** Bo-Sung Kim, Bosung Kim, Seyeong Yoon, Wonyoung Park, Sung-Jin Bae, Jongkil Joo, Wonnam Kim, Ki-Tae Ha

**Affiliations:** 1 Research Institute for Korean Medicine, Pusan National University, Yangsan 50612, Republic of Korea; kkc0704@pusan.ac.kr (B.-S.K.); jinling0122@pusan.ac.kr (W.P.); 2 Department of Korean Medical Science, School of Korean Medicine, Pusan National University, Yangsan 50612, Republic of Korea; kbs0912@pusan.ac.kr (B.K.); ysy9641@gmail.com (S.Y.); 3 Department of Molecular Biology and Immunology, Kosin University College of Medicine, Busan 49267, Republic of Korea; dr.baesj@kosin.ac.kr; 4 Department of Obstetrics and Gynecology, School of Medicine, Pusan National University Hospital, Busan 49241, Republic of Korea; jkjoo@pusan.ac.kr; 5 Division of Pharmacology, School of Korean Medicine, Pusan National University, Yangsan 50612, Republic of Korea

**Keywords:** endometriosis, metabolic reprogramming, Warburg’s effect, aerobic glycolysis mitochondrial dysfunction

## Abstract

Endometriosis is a chronic gynecological disorder characterized by the presence of endometrial-like tissue outside the uterus, leading to inflammation, pain, and infertility. Emerging evidence indicates that endometriotic lesions exhibit cancer-like properties, including metabolic reprogramming marked by increased glucose uptake, enhanced Warburg’s effect, and altered mitochondrial function. These metabolic adaptations support cell survival under hypoxic conditions and contribute to immune evasion and sustained proliferation. This review summarizes current findings on the molecular mechanisms driving metabolic reprogramming in endometriosis, including the roles of mitochondrial dysfunction, hypoxia-inducible factor (HIF) signaling, the PI3K/AKT/mTOR pathway, inflammatory cytokines, and genetic and epigenetic regulators. In addition, we discuss therapeutic strategies targeting glycolytic pathways using both synthetic inhibitors and natural compounds, which represent promising non-hormonal options. Finally, we highlight the need for further preclinical and clinical studies to validate metabolic interventions and improve outcomes for patients with endometriosis.

## 1. Introduction

Endometriosis is a chronic gynecological condition characterized by the presence of endometrial-like tissue outside the uterus, mainly in the ovaries, fallopian tubes, and peritoneum [1]. This ectopic endometrium undergoes cyclic changes similar to the normal endometrium, causing inflammation, fibrosis, and adhesion formation. Endometriosis is estimated to affect 10–15% of reproductive age women with severe health problems, such as chronic pelvic pain, dysmenorrhea, dyspareunia, and infertility [2,3]. Despite its high prevalence, the precise pathogenesis of this disease has not yet been fully elucidated [4,5]. Several factors, including hormonal imbalance, immune dysfunction, genetic predisposition, and environmental influences, contribute to the development and progression of this disease. Due to the non-specific symptoms and limited public recognition, many patients experience delays in diagnosis and treatment. Current treatment options involve surgical and hormonal therapies, which focus on symptom management rather than fundamental treatment [6,7]. Since hormone therapy is often accompanied by serious side effects, the need for non-hormonal treatments has been in the spotlight [8].

Endometriosis manifests several cancer-like characteristics, including local invasion, distant metastasis, apoptotic cell death resistance, and immune evasion [9]. Recent evidence shed light on shared pathological mechanisms of the two diseases; a process known as metabolic reprogramming is one of the key mechanisms. Specifically, endometriotic lesions also exhibit a metabolic profile similar to the Warburg’s effect in cancer cells, which is characterized by increased glucose uptake, enhanced glycolysis, and altered mitochondrial function [10]. The metabolic shift to aerobic glycolysis promotes energy production and the biosynthesis of cellular building blocks, which helps the survival and proliferation of ectopic endometrial cells [11]. The Warburg’s effect enables migrated endometrial cells to adapt to the low-oxygen environment of the peritoneal cavity, avoiding cell death and maintaining an inflammatory condition. Therefore, targeting glycolytic metabolism—a key pathway underlying the Warburg’s effect—emerges as a promising therapeutic strategy for endometriosis.

This review provides a detailed overview of the molecular mechanisms underlying aerobic glycolysis in endometriosis, along with therapeutic approaches, including metabolic inhibitors and dietary interventions. A comprehensive understanding of these metabolic features is expected to facilitate the development of more effective treatment strategies and ultimately improve clinical outcomes.

## 2. Overview of the Warburg’s Effect

The Warburg’s effect, first described by German physician and biochemist Otto Warburg in 1923, describes the metabolic reprogramming of cancer cells in which glucose metabolism is switched from oxidative phosphorylation to aerobic glycolysis, even when sufficient oxygen is present [12]. Normal cells produce ATP mainly through mitochondrial oxidative phosphorylation, while cancer cells prefer glycolysis and convert pyruvate to lactic acid without entering the tricarboxylic acid (TCA) cycle. This phenomenon provides a number of benefits that promote tumor growth, including rapid production of ATP, availability for biosynthetic building blocks, and adaptation to hypoxic environments [13]. It was selected as one of the 10 hallmarks of cancer in Douglas Hanahan’s landmark article “Hallmarks of Cancer: The Next Generation” in 2011 [14].

**Figure 1:**
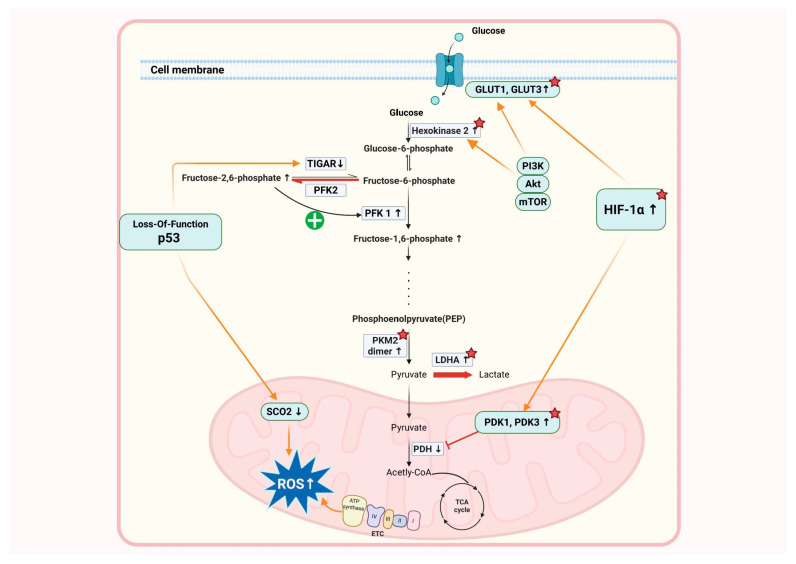
Molecular and metabolic pathways underlying the Warburg’s effect in cancer. Cancer cells preferentially utilize aerobic glycolysis over mitochondrial oxidative phosphorylation even in the presence of oxygen, a phenomenon known as the Warburg effect. This metabolic reprogramming is regulated by several oncogenic and tumor-suppressive pathways. HIF-1α, stabilized under hypoxic or oncogenic conditions, promotes the expression of GLUT1 and GLUT3 and PDK1 and PDK3, which inhibit PDH, thereby blocking the entry of pyruvate into the TCA cycle. The PI3K/AKT/mTOR pathway enhances glycolysis by upregulating GLUT1 and HK2 while supporting cell proliferation and survival. Conversely, the tumor suppressor p53 inhibits glycolysis via TIGAR and enhances oxidative phosphorylation through SCO_2_. Loss of p53 in tumors removes these inhibitory mechanisms, allowing glycolysis to proceed unchecked. Cancer cells also exhibit metabolic plasticity, adapting between glycolysis and oxidative phosphorylation, depending on the environmental conditions, and frequently increase glutamine metabolism to fuel the TCA cycle. These adaptations not only support biosynthesis and rapid growth but also promote invasion, metastasis, and immune evasion, contributing to therapy resistance. The red asterisks indicate the molecular targets of therapeutic drugs.

As illustrated in Figure 1, the Warburg’s effect is facilitated at the molecular level by the upregulation of key glycolytic enzymes and the activation of oncogenic signaling pathways. Many cancer cells overexpress hexokinase 2 (HK2), which catalyzes the first step of glycolysis by phosphorylating glucose and trapping it in the cell [15]. Another key regulator is pyruvate kinase M2 (PKM2), which is an isozyme of the less active pyruvate kinase that exists in a dimeric form in cancer cells [16]. This altered enzyme activity slows down the conversion of phosphoenolpyruvic acid to pyruvic acid, resulting in the accumulation of intermediates that serve as precursors for anabolic pathways involved in nucleotide, amino acid, and lipid biosynthesis. In addition, increased expression of lactate dehydrogenase A (LDHA) promotes the conversion of pyruvate to lactate, facilitating a continuous flow of glycolysis by regenerating NAD⁺. The Warburg’s effect is closely related to tumor-promoting signaling pathways that regulate metabolism [17]. An essential transcription factor, hypoxia-inducible factor-1α (HIF-1α) is stabilized in hypoxic environments but is upregulated in many cancers regardless of oxygen availability. HIF-1α induces the expression of various glycolysis-related proteins, including glucose transporters (GLUT1 and GLUT3), and pyruvate dehydrogenase kinase 1 (PDK1) and 3 (PDK3). PDK inhibits the activity of pyruvate dehydrogenase (PDH), thereby preventing pyruvate from being converted to acetyl-CoA and entering the TCA cycle. Another important signaling cascade is the PI3K/AKT/mTOR pathway [18]. This pathway upregulates GLUT1 and HK2 to enhance glucose uptake and glycolysis while inducing cell survival and proliferation. Furthermore, the oncogene MYC plays an important role in enhancing the metabolic reprogramming of cancer cells by activating the production of glycolytic enzymes and mitochondrial biogenesis [19].

The Warburg’s effect is caused by both active oncogenes and the suppression of tumor suppressor genes. The tumor suppressor gene p53 is frequently mutated in cancer and plays an important role in metabolic regulation [20,21]. Wild-type p53 negatively regulates glycolysis by inducing TP53-induced glycolysis and apoptosis regulator (TIGAR), which lowers the level of fructose-2,6-phosphate, a key activator of phosphofructokinase-1 (PFK1) [22]. p53 also enhances mitochondrial respiration by promoting the expression of cytochrome c oxidase 2 (SCO_2_), an important component of the electron transport chain [23]. When the p53 function is lost in cancer cells, these constraints disappear, allowing glycolysis to proceed unchecked and metabolic adaptation to be further enhanced.

The Warburg’s effect is often related to mitochondrial dysfunction, although cancer cells do not completely abandon oxidative phosphorylation [24]. Instead, many tumors exhibit metabolic plasticity, utilizing both glycolysis and mitochondrial metabolism, depending on the environmental conditions [25]. This flexibility allows cancer cells to survive in fluctuating oxygen levels and nutrient availability. Also, glutamine metabolism is often increased in tumors, supplying α-ketoglutarate to the TCA cycle and supporting the biosynthesis pathways necessary for rapid proliferation [26]. The interplay between these processes and alternative metabolic pathways highlights the complexity of cancer metabolism that extends well beyond energy production to include cell signaling, redox balance, and immune evasion.

Metabolic reprogramming observed in cancer cells has a significant impact on tumor progression. Increased glucose uptake and lactate production promote invasion and metastasis while contributing to the formation of an acidic microenvironment that suppresses immune cell activity [27,28]. In addition, the conversion to aerobic glycolysis enables the rapid production of macromolecules required for cell division, which is beneficial for cancer cell growth [29]. Moreover, it can produce ATP quickly without the increase in reactive oxygen species (ROS) that inevitably occurs in mitochondrial respiration using oxygen. The ability of cancer cells to re-adjust their metabolism as described above enables them to resist apoptosis and evade immune surveillance, which increases the complexity of treatment strategies by causing resistance not only to conventional chemotherapeutics but also immunotherapies.

Understanding the molecular mechanisms underlying the Warburg’s effect can provide valuable insights into the metabolic vulnerability of cancer cells. Aerobic glycolysis is a hallmark of tumor metabolism; however, the extensive metabolic network that supports tumor growth is highly dynamic, influenced by genetic mutations, tumor microenvironmental factors, and immune interactions. Unraveling these complex metabolic dependencies presents potential opportunities for cancer therapy.

## 3. Metabolic Reprogramming in Endometriosis

**Figure 2:**
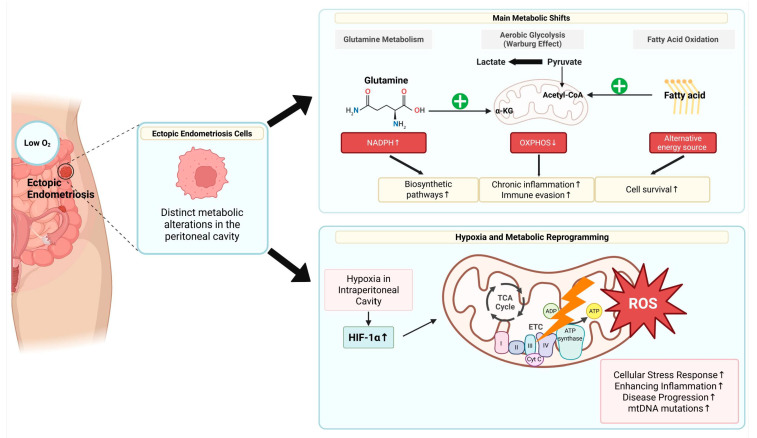
Metabolic reprogramming in endometriosis: A shift toward glycolysis and mitochondrial dysfunction. Ectopic endometriotic cells exhibit distinct metabolic alterations compared to normal endometrial cells, including a shift toward aerobic glycolysis (Warburg effect), increased fatty acid oxidation, and mitochondrial dysfunction. Hypoxia in the peritoneal cavity activates HIF-1α, which enhances glycolytic gene expression and suppresses oxidative phosphorylation. These changes result in elevated lactate production, promoting an acidic and inflammatory microenvironment that facilitates immune evasion and lesion survival. Enhanced fatty acid oxidation provides alternative energy under stress, while glutamine metabolism supports NADPH production and biosynthesis. Mitochondrial dysfunction, characterized by impaired electron transport, increased ROS, and mtDNA mutations, further drives disease progression.

Recent research has highlighted metabolic reprogramming as a key factor in the pathophysiology of endometriosis, influencing cell survival, proliferation, and immune evasion [10,30,31,32]. In normal endometrial cells, the metabolic process is tightly regulated by the hormonal cycle. However, ectopically located endometriotic cells in the peritoneal cavity exhibit distinct metabolic alterations that distinguish them from normal cells (Figure 2) [33,34]. A characteristic of this change is the shift to aerobic glycolysis, commonly known as the Warburg’s effect, in which the pathway is re-routed so that glucose metabolism prefers glycolysis rather than oxidative phosphorylation (OXPHOS). Increases in lactic acid production lead to an acidic microenvironment that promotes inflammation and immune evasion. In addition, fatty acid oxidation is increased in endometriotic lesions, providing an alternative energy source to maintain cell survival under stress conditions [35]. Glutamine metabolism has also been reported to contribute to the production of NADPH, which is essential for cell proliferation, and to the biosynthesis process [30,36]. Hypoxia, a common feature of endometriotic lesions, plays an important role in promoting these metabolic changes [37,38]. Since the intraperitoneal cavity, where endometriosis develops, has a lower oxygen concentration than normal tissues, HIF-1α is activated under hypoxic conditions, which, in turn, inhibits mitochondrial oxidative phosphorylation and enhances gene expression of the glycolysis pathway. As a result, mitochondrial dysfunction—characterized by decreased efficiency of the electron transport chain, increased ROS production, and mitochondrial DNA mutations—is frequently observed in endometriosis cells. These changes contribute to the cellular stress response, further enhancing inflammation and disease progression [39]. Initially, this change was understood to be simply an adaptation to the intraperitoneal environment with a shortage of oxygen; however, a series of studies conducted in the late 2010s showed that cancer-like genetic mutations appear in endometrial tissue and accumulate further in ectopic endometrial tissue [40,41]. Therefore, it has been proposed that the accumulation of these genetic mutations may lead to metabolic abnormalities in endometriotic cells.

Metabolic reprogramming in endometriosis, as represented in Figure 2, represents a multifaceted process involving increased glycolysis, enhanced fatty acid oxidation, and mitochondrial dysfunction, all contributing to the persistence and progression of the disease [42]. Understanding these metabolic adaptations may open new avenues for potential therapeutic interventions targeting specific metabolic pathways to alleviate the burden of endometriosis.

## 4. Molecular Mechanisms Underlying Warburg-like Metabolic Reprogramming

### 4.1. Hypoxia-Inducible Factor (HIF) Signaling and Glycolysis Activation

**Figure 3:**
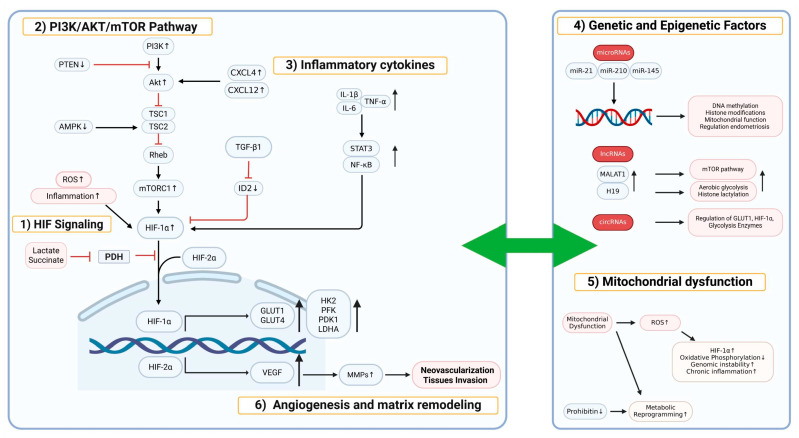
Glycolysis-related metabolic changes, driven by genetic, epigenetic, and mitochondrial dysfunction, underlie endometriosis pathogenesis. Endometriotic cells undergo extensive metabolic reprogramming driven by hypoxia, inflammatory cytokines, genetic/epigenetic alterations, and mitochondrial dysfunction. Hypoxia in the peritoneal environment stabilizes HIF-1α and HIF-2α, enhancing glycolysis by upregulating GLUT1; GLUT4; glycolytic enzymes (HK2, PFK, and LDHA); and PDK1 while also promoting angiogenesis via VEGF. The PI3K/AKT/mTOR signaling cascade, often activated by PTEN loss or PIK3CA mutation, further boosts HIF-1α expression and glycolytic metabolism. Chronic inflammation involving TGF-β1, IL-6, IL-1β, and TNF-α activates NF-κB and STAT3 pathways, reinforcing the glycolytic phenotype and suppressing oxidative phosphorylation. Additionally, chemokines such as CXCL12/CXCR4 contribute to metabolic and migratory changes. Genetic mutations and epigenetic modifications (e.g., miRNAs, lncRNAs, and DNA methylation) influence the expression of key metabolic regulators. Mitochondrial dysfunction—evidenced by reduced oxidative phosphorylation enzyme expression, PHB loss, and excess ROS—contributes to HIF-1α stabilization, inflammation, and enhanced proliferation. These metabolic changes promote angiogenesis and ECM remodeling through increased lactate production, VEGF expression, and MMPs, ultimately driving lesion persistence and invasiveness.

Hypoxia is a characteristic feature of endometriotic lesions, which is caused by the nature of the peritoneal environment, insufficient vascularization of ectopic endometrial tissue, and high metabolic demand [37]. Under these conditions, HIF-1α is stabilized and translocated to the nucleus, likely avoiding proteasome degradation. HIF-1α activates the transcription of genes essential for survival and glycolysis under oxygen deprivation. McKinnon et al. explained that this activation increases the expression of glucose transporters such as GLUT1 and GLUT4, enabling a continuous supply of glucose (Figure 3) [43]. HIF-1α also increases the expression of important glycolytic enzymes, including HK2, PFK, PDK1, and LDHA [44]. Several researchers have further emphasized that TGF-β1 and hypoxia increase the activity of HIF-1α, leading to the enhancement of the process [10,45,46,47]. In addition, hypoxia-induced stabilization of HIF-2α and its interaction with the VEGF pathway promote angiogenesis and establish a positive feedback loop that exacerbates hypoxic conditions and enhances pathological processes such as neovascularization and tissue invasion [48,49]. Recently, non-canonical mechanisms have been proposed in which HIF is activated by metabolic products such as lactate and succinate even when the tissue is not actually hypoxic, indicating that these mechanisms may also affect endometriosis [50]. Prior research showed that the buildup of ROS completes the pathological cycle and supports the growth of the lesion by stabilizing HIF-1α and promoting inflammation [51,52,53]. In particular, oxidative stress and reactive oxygen species (ROS) production are both a consequence and a driving force of glycolytic reprogramming. Accumulated ROS can activate key signaling pathways such as HIF-1α and NF-κB, which, in turn, enhance the expression of glycolytic enzymes and glucose transporters, thereby reinforcing the glycolytic phenotype [54,55]. Conversely, the shift toward aerobic glycolysis reduces mitochondrial oxidative phosphorylation, which can lead to electron leakage and further ROS generation [56]. This bidirectional relationship contributes to a self-sustaining cycle that supports the survival and proliferation of endometriotic cells under hypoxic conditions.

### 4.2. PI3K/AKT/mTOR Pathway and Metabolic Changes

A number of studies have shown that the PI3K/AKT/mTOR pathway is deeply involved in the metabolic reprogramming of endometriosis [57,58]. As presented in Figure 3, activation of PI3K induces downstream phosphorylation of AKT and activation of mTORC1, which, in turn, enhances HIF1A expression and glycolytic enzyme activity. The mTORC1 complex not only promotes glycolysis but also protein synthesis, lipid biosynthesis, and nucleotide production to meet the metabolic demands of rapidly dividing cells. Crosstalk with AMP-activated protein kinase (AMPK) and feedback regulation through Tuberous sclerosis complex 2 (TSC2) ensure metabolic balance, whereas persistent mTOR activation in endometriotic lesions results in uncontrolled glycolysis and cell proliferation [59]. Additional evidence from protein and transcriptome studies demonstrates that phosphatase and tensin homolog deleted on chromosome 10 (PTEN) loss and AKT hyperactivation lead to metabolic rearrangements that contribute to extracellular matrix remodeling, angiogenesis, and invasiveness [60,61,62]. Furthermore, PIK3CA may be involved in encouraging the glycolysis of endometriosis, because it is one of the cancer-like mutations discovered in endometriotic tissue [63].

### 4.3. Role of Inflammatory Cytokines and Metabolic Changes

Chronic inflammation is a distinctive feature of endometriosis, and inflammatory cytokines directly affect cellular metabolism. Young et al. (2014, 2016) demonstrated that transforming growth factor-β1 (TGF-β1) regulates inhibitor of DNA-binding 2 (ID2), which, in turn, releases HIF-1α from inhibition and enhances the process [10,64]. In addition, cytokines such as interleukin (IL)-6, IL-1β, and tumor necrosis factor-α (TNF-α) activate the nuclear factor kappa B (NF-κB) and signal transducer and activator of transcription 3 (STAT3) pathways, which upregulate glycolytic enzymes and downregulate mitochondrial oxidative phosphorylation (Figure 3) [65,66]. Several studies have confirmed that inflammatory cytokines promote glucose uptake and lactate production in endometriotic cells [67,68]. The studies also revealed that chemokines such as CXCL12 and its receptor CXCR4 play an important role in regulating glucose metabolism and cell migration [69,70]. Furthermore, several investigations have provided transcriptomic and proteomic evidence that inflammatory signals induce metabolic adaptation by inhibiting mitochondrial oxidative phosphorylation while simultaneously upregulating certain mitochondrial and glycolytic enzymes involved in alternative metabolic routes [71,72]. This seemingly paradoxical regulation reflects a cellular compensatory mechanism; while mitochondrial respiration is suppressed due to hypoxia or inflammation-induced damage, cells increase the expression of enzymes related to mitochondrial biogenesis, antioxidant defense (e.g., SOD2), or metabolic flexibility (e.g., citrate synthase and PDK1) to adapt to stress and sustain energy production through glycolysis and other non-canonical pathways. Thus, the upregulation of these enzymes does not necessarily indicate enhanced mitochondrial activity but rather a shift in metabolic programming to cope with mitochondrial dysfunction [73,74,75].

### 4.4. Genetic and Epigenetic Factors Contributing to Metabolic Reprogramming

Epigenetic regulation and genetic predisposition contribute significantly to metabolic rearrangement in endometriosis. According to whole-genome studies and epigenomic profiling, endometriosis lesions adapt through stable genetic and epigenetic modifications [76,77,78]. Genomic modifications such as single-nucleotide polymorphisms in metabolic genes and mutations in regulators of the PI3K/AKT/mTOR pathway can induce metabolic reprogramming. Several studies have emphasized that DNA methylation changes, histone modifications, and regulation by microRNAs such as miR-21, miR-210, and miR-145 affect the pathways and mitochondrial function (Figure 3) [79,80,81]. In addition to miRNAs, it has been found that some lncRNAs, such as H19 and MALAT1, and some circRNAs play an important role in the regulation of glycolysis [82,83,84,85]. Wen et al. [86] highlighted key lncRNAs that promote the expression of glycolytic enzymes and affect glucose metabolism in endometriotic cells.

### 4.5. The Role of Mitochondrial Dysfunction in the Progression of Endometriosis

Mitochondrial dysfunction is an important factor in the metabolic reprogramming of endometriotic cells. Several researchers have demonstrated that the reduction of prohibitin (PHB) and mitochondrial dysfunction increases the dependence on metabolic reprogramming (Figure 3) [87,88,89]. In addition, proteomic changes were identified that are consistent with mitochondrial dysfunction. For instance, mitochondrial dysfunction is associated with the downregulated expression of key enzymes in the oxidative phosphorylation pathway [90,91,92]. The increase in ROS generated by dysfunctional mitochondria contributes to genomic instability and chronic inflammation. Moreover, mitochondrial dysfunction has been linked to endometriotic cell proliferation and invasion of the surrounding tissues through ROS-stabilized HIF-1α [93,94].

### 4.6. Interaction with Angiogenesis and Matrix Remodeling

Angiogenesis and remodeling of the extracellular matrix (ECM) are closely related to metabolic changes (Figure 3). Increased lactate production and secretion promote VEGF expression and neovascularization [95,96]. Several studies have reported that increased matrix metalloproteinases (MMPs), by the glycolysis signal, allows the invasion into neighboring tissues [97,98].

## 5. Current Research on Glycolysis Inhibitors in Endometriosis

**Figure 4:**
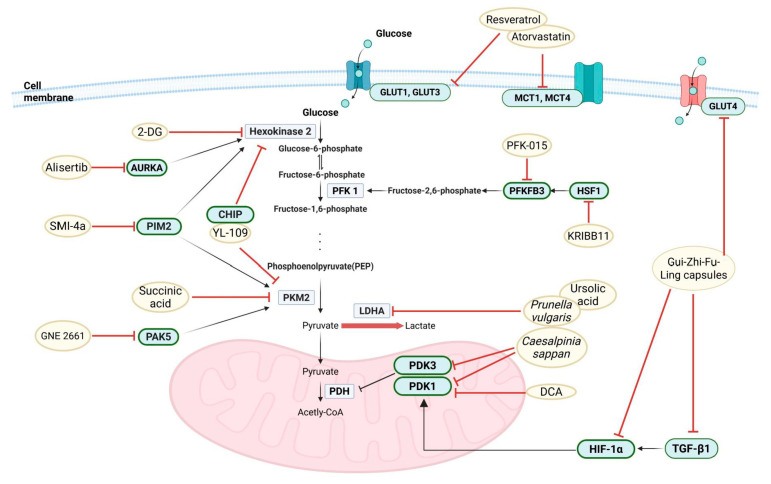
Overview of the molecular targets and glycolysis inhibitors under investigation for endometriosis therapy. Multiple molecular targets and candidate compounds have been investigated to inhibit aerobic glycolysis in endometriotic cells. PDK1 and PDK3 are the most studied targets, with DCA being the leading compound shown to restore mitochondrial function, reduce lactate production, and alleviate pain. Natural extracts such as *Caesalpinia sappan* and *Prunella vulgaris* inhibit PDKs and LDHA, reducing glycolysis and promoting apoptosis. Other notable targets include PKM2, inhibited by succinic acid and PIM2 inhibitors (e.g., SMI-4a), and PAK5, which inhibition destabilizes PKM2 and suppresses glycolysis. PFKFB3 is targeted by PFK-015 and regulated by HSF1, which is inhibited by KRIBB11. The inhibition of HK2 with 2-DG also suppresses glycolysis and macrophage infiltration. Additional compounds like alisertib (AURKA inhibitor), YL-109 (CHIP agonist), and natural agents such as resveratrol and Gui-Zhi-Fu-Ling capsules show multi-targeted effects, including anti-glycolytic, anti-fibrotic, and anti-angiogenic actions.

As shown in Table 1 and illustrated in Figure 4, ongoing research on glycolysis inhibitors for endometriosis is being conducted using various compounds, depending on the molecular target. A brief comparison of key candidate compounds is summarized in Table 2. The PDK family has emerged as the most important target, and dicarboxylate (DCA), a drug that was long ago approved by the FDA as an inhibitor of lactic acidosis, is the most extensively studied candidate. In previous preclinical and early clinical studies (Horne et al. 2019; Lee HC et al. 2019; Leow HW et al. 2021), DCA was shown to restore mitochondrial function by inhibiting PDK1, reduce lactic acid production, and alleviate endometriosis-related pain [99,100,101]. Similarly, *Caesalpinia sappan* extract was shown to inhibit the glycolytic activity of endometriotic cells and induce apoptosis by reducing the expression of PDK1 and PDK3 [102]. However, it should be noted that DCA requires relatively high concentrations (in the millimolar range) to exert its effects, which raises concerns about specificity and potential off-target effects. Despite this limitation, its progression to early-phase clinical trials highlights its significance in the field and underscores the need for further research into more potent and selective metabolic inhibitors. Moreover, enzymes like LDHA and PKM2 are garnering attention [103]. LDHA inhibition through shRNA has been shown to suppress glycolysis and promote cell death in endometriotic cells [104]. In addition, Cho et al. (2022) reported that *Prunella vulgaris* extract effectively reduces aerobic glycolysis and prevents lesion growth by inhibiting LDHA as the main target and inhibiting the expression of PDK1 and PDK3 at the same time [105]. They also showed that ursolic acid, a constituent of *Prunella vulgaris*, effectively inhibits LDHA [106]. Recently, a number of research groups have been actively conducting research on LDHA inhibitors with anticancer activity, so it is expected that research on these drugs for endometriosis will also be expanded. Research on PKM2 has revealed that inhibiting PKM2 using succinic acid reduces cell viability by inhibiting the corresponding process in endometriosis stromal cells [107]. Proviral insertion in murine lymphomas 2 (PIM2) kinase has been identified as a key regulator of the expression of glycolysis enzymes such as PKM2 and HK2, while inhibiting PIM2 by treating SMI-4a reduces glycolysis and fibrosis [108,109]. In addition, p21-activated kinase 5 (PAK5) increases the protein stability of PKM2; thus, inhibiting PAK5 with GNE 2661 inhibited glycolysis and growth in endometriosis [110].

Another notable target is phosphofructokinase/fructose bisphosphatase (PFKFB3), and the inhibitor PFK-015 has been shown to inhibit cell proliferation, migration, and invasion by suppressing the glycolysis process through the inhibition of the enzyme at the cellular and in vivo levels [117]. Recent studies have confirmed that heat shock factor 1 (HSF1) regulates the expression of PFKFB3 and that the inhibitor KRIBB11 suppresses endometriosis in animal experiments [113,114]. Glucose analog 2-DG inhibits HK2, the most upstream enzyme of the glycolysis pathway. It was verified that 2-DG suppressed the infiltration of differentiated M2 macrophages in the endometriotic lesions [121]. In the study, it was found that the Mettl3/Trib1/ERK-STAT3 pathway plays an important role [116].

The involvement of Aurora kinase A (AURKA) and carboxyl terminus of the Hsc70-interacting protein (CHIP) pathways in glycolysis regulation provides new therapeutic targets. Alisertib (AURKA inhibitor) and YL-109 (CHIP agonist) have shown potential for the treatment of endometriosis in animal studies [115,118]. Resveratrol, a natural product found in grapes, has demonstrated inhibitory effects on glycolysis and anti-angiogenesis in animal models when administered alone or in combination with atorvastatin [112]. Gui-Zhi-Fu-Ling capsules, a traditional Chinese medicine, have also shown a positive effect on endometriosis by inhibiting multiple targets such as TGF-β1, GLUT4, and VEGF [111,122].

However, despite the accumulation of research on the metabolic abnormality of endometriosis, especially aerobic glycolysis, more preclinical and clinical research needs to be carried out. Most animal experiments using molecular targeted inhibitors are still not enough to be considered as accurate preclinical research. To date, the only clinical trial targeting the glycolytic pathway of endometriosis has found that DCA could suppress pain in endometriosis patients. We expect that research on drug development using diverse molecular targets in this field will become increasingly active in the future.

## 6. Clinical Implications of Metabolic Reprogramming in Endometriosis

**Figure 5:**
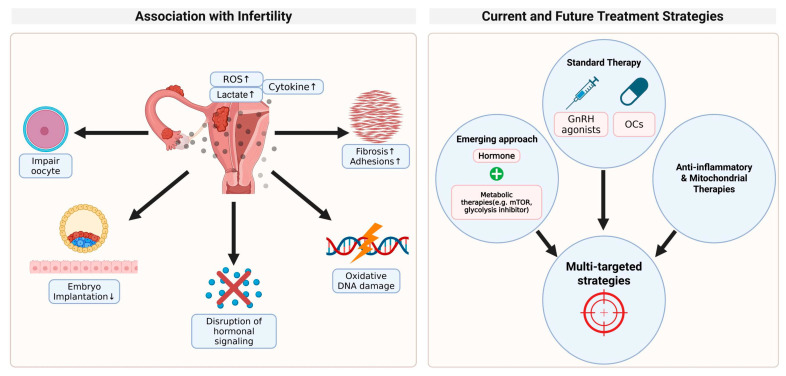
Role of metabolic reprogramming in endometriosis pathophysiology and therapeutic strategies. The shift to glycolysis in endometriotic lesions increases lactate production, creating an acidic microenvironment that promotes angiogenesis, tissue invasion, and immune evasion. These changes lead to chronic inflammation, extracellular matrix remodeling, and increased nerve fiber density, contributing to pain and symptom severity. Metabolic alterations also impair fertility by affecting the oocyte quality and endometrial receptivity. While hormonal therapies target estrogen-driven proliferation, combining them with metabolic inhibitors like mTOR and glycolysis blockers may improve treatment outcomes. Anti-inflammatory and mitochondrial therapies could further reduce recurrence. However, most of the evidence is preclinical, underscoring the need for more clinical studies.

Metabolic reprogramming, especially the shift to glycolysis, plays an important role in the persistence and progression of endometriotic lesions. Increased lactate production and altered energy metabolism create an acidic microenvironment that promotes angiogenesis, tissue invasion, and immune evasion [119,120]. These metabolic abnormalities lead to changes in the extracellular matrix composition and the immune cell infiltration. This metabolic state not only helps the survival of ectopic endometrial cells but also promotes chronic inflammation, which increases the severity of symptoms (Figure 5). The continuous metabolic flux can alter the local nerve fiber density and contribute to the development of neuropathic pain [10,123]. In particular, pain is the most critical symptom that reduces the quality of life of patients with endometriosis. In clinical studies, PDK1 inhibitor DCA has been confirmed to contribute to the reduction of pain in endometriosis patients.

Inflammatory responses affecting the oocyte, the endometrium, and the hormonal regulation are closely associated with infertility in endometriosis patients [124]. High lactate levels and oxidative stress can reduce the quality of eggs, interfere with the receptivity of the endometrium, and affect embryo implantation [125]. Metabolic environment change can disrupt hormonal signaling in reproductive tissues and cause oxidative DNA damage [126,127]. In addition, the accumulation of inflammatory cytokines and neurovascular factors in the active lesion of glycolysis can cause chronic pelvic pain and dysmenorrhea [128]. The pain pathway is further sensitized by local nerve growth, cytokine release, and metabolic byproducts such as lactic acid and reactive oxygen species. Fibrosis is promoted by altered metabolites, and inflammatory cytokines induce serious complications, including adhesions to the surrounding tissue of endometriosis lesions.

Currently, the standard drug treatment for endometriosis, hormonal therapy (e.g., GnRH agonists and oral contraceptives), aims to suppress estrogen-induced proliferation, although it does not directly act on metabolic changes (Figure 5) [129,130]. Recent evidence suggests that the combination of hormonal therapy and metabolic interventions may improve treatment outcomes. For example, mTOR inhibitors and glycolysis inhibitors can improve the effectiveness of standard treatments by addressing the metabolic dependency of endometriosis lesions. Metabolic therapy can help overcome treatment resistance in patients with active metabolic lesions. Furthermore, anti-inflammatory and mitochondrial-targeted therapies can complement existing therapies to reduce recurrence rates and provide more effective symptom relief. However, it is worth noting that most of the supporting studies for these metabolic interventions are limited to preclinical research, with only one small-scale clinical trial conducted to date. As such, the current evidence base remains insufficient, underscoring the need for further clinical investigations to validate these promising therapeutic strategies.

## 7. Conclusions and Future Perspectives

Warburg-like metabolic reprogramming is one of the fundamental features of endometriosis, contributing to lesion survival, progression, and symptom severity. Under hypoxic and inflammatory conditions, the transition to this process promotes angiogenesis, immune evasion, and tissue invasion while also exacerbating pain and infertility. Understanding these metabolic changes has led to new treatment methods that target metabolic vulnerabilities in endometriotic lesions, as well as hormonal pathways. It is expected that metabolic inhibitors, when combined with existing treatments, will enhance treatment efficacy, reduce recurrence, and improve patients’ quality of life. As research continues, we believe that metabolic profiling and precision medicine approaches will play an increasingly important role in the clinical management of endometriosis.

Future molecular studies of the Warburg’s effect in endometriosis should focus on several key areas. First, the precise regulatory mechanisms of HIF-1α and HIF-2α activation under fluctuating hypoxic conditions in the endometriotic microenvironment should be elucidated. It is important to understand how these factors interact with inflammatory cytokines and hormonal signaling. Second, the role of mitochondrial dynamics, including mitophagy and biogenesis, in glycolytic reprogramming maintenance requires further investigation. Third, studies should explore the post-transcriptional regulation of glycolytic enzymes and transporters, with a focus on non-coding RNAs such as lncRNAs and circRNAs. Fourth, the development of advanced metabolomics and single-cell transcriptomics technologies will enable the identification of subpopulations of endometriotic cells with distinct metabolic phenotypes, which will allow for personalized treatment. Finally, large-scale translational studies are needed to validate the efficacy of metabolic biomarkers for early diagnosis, treatment monitoring, and prognosis prediction.

This area of research will help to deepen our understanding of metabolic changes and develop new therapeutic strategies for endometriosis. Future studies will likely explore the use of combination metabolic inhibitors and non-hormonal therapies in patient populations that do not respond to or are contraindicated for hormonal therapy. Clinical trials focused on metabolic regulation are poised to change the landscape of endometriosis treatment and have the potential to improve long-term outcomes and quality of life for affected women.

## Figures and Tables

**Table 1:** Summary of the articles retrieved from PubMed using the terms “glycolysis” and “endometriosis”.

First Author	Key Mechanism	Molecular Target	Drug	Experiments	Publication Year	Ref
McKinnon B	Altered glucose metabolism is mediated by glucose transporter	GLUT4	-	In vitro, In vivo	2014	[43]
Young VJ	TGF-β induces a Warburg-like effect	HIF1A, PDK1, LDHA, GLUT1	-	In vitro, Human sample	2014	[10]
Qi X	Enhanced glycolysis is due to prohibitin (PHB) downregulation	PHB	-	In vitro, Human sample	2014	[87]
Kasvandik S	Warburg effect leads to enhanced invasiveness, adhesion, and immune evasion.	-	-	Human sample, In silico	2016	[75]
Young VJ	TGF-β1-induced Warburg effect via suppression of ID2, which in turn upregulates HIF-1α expression.	ID2	-	In vitro, Human sample	2016	[64]
Zhou J	Regulation of glycolysis and gluconeogenesis leads to metabolic changes and modulation of TGF-β1, GLUT-4, and VEGF expression	TGFB1, GLUT4, VEGF	Gui-Zhi-Fu-Ling Capsules	In vivo, In silico	2018	[111]
Lee HC	Hypoxia upregulates PDK1 to enhance glycolysis	PDK1	Dichloroacetate	In vitro	2019	[99]
Horne AW	TGF-β1 drives increased glycolysis, reduced mitochondrial respiration, and enhanced lactate production.	PDK1	Dichloroacetate	In vitro, In vivo, Human sample	2019	[100]
Rytkönen KT	Hypoxia-driven transcription factors Jun/Fos and CEBP, alters glycolysis, epithelial-mesenchymal transition, and inflammatory pathways.	Jun, Fos, CEBP	-	In vitro, Human sample	2020	[39]
Pocate-Cheriet K	Metabolic reprogramming in follicular fluid is characterized by altered glycolysis, beta-oxidation, and mitochondrial dysfunction.	-	-	Human sample, In silico	2020	[42]
Bahrami A	Inhibition of glycolysis and neovascularization suppresses endometriosis development.	GLUT1, GLUT3, MCT1, MCT4	Atorvastatin and Resveratrol	In vivo	2021	[112]
Leow HW	Metabolic reprogramming correction reduces endometriosis-associated pain.	PDK1	Dichloroacetate	Human clinical trials	2021	[101]
Kim BS	Inhibition of aerobic glycolysis and induction of ROS-mitochondria-mediated apoptosis.	PDK1, PDK3	*Caesalpinia sappan*	In vitro	2021	[102]
Wang Y	HSF1 promotes glycolysis via upregulating PFKFB3 facilitates endometriosis progression.	HSF1, PFKFB3	KRIBB11 (HSF1 inhibitor)	in vitro, In vivo	2021	[113]
Zheng J	LDHA promotes glycolysis and inhibits mitochondrial function.	LDHA	shLDHA	In vitro, Human sample	2021	[104]
Yao Q	PKM2 inhibition and may serve as a potential treatment for endometriosis.	PKM2	Cinnamic acid	In vitro, Human sample	2021	[107]
Cho MK	Inhibition of aerobic glycolysis prevents lesion growth and metabolic adaptation.	LDHA, PDK1, PDK3	*Prunella vulgaris*	In vitro, In vivo	2022	[105]
Hou S	Downregulation of HK2 reduces migration, invasion, and proliferation of endometrial stromal cells.	HK2, STAT1	-	In vitro	2022	[11]
Mao J	Upregulation of PHB1 enhances glucose metabolism, ATP synthesis, and ROS production.	PHB1	-	In vitro, Human sample	2022	[88]
Wang H	FTO regulates ATG5 expression through m6A methylation and in turn, suppresses the expression of PKM2.	FTO, ATG5, PKM2	-	In vitro, Human sample	2022	[103]
Lu C	The PIM2-PFKFB4 axis drives glycolysis and cell growth, contributing to EM progression.	PIM2, PFKFB4	-	In vitro, In vivo, Human sample	2022	[109]
Li L	Three key feedback loops were discovered in the TF-miRNA-hub gene network	MYC, YY1, HIF1A, LDHA, RELA, miR-34a-5p, miR-155-5p, miR-93-5p	-	Human sample, In silico	2022	[85]
Ling X	OTUB1 increase the HSF1 stability and enhances glycolysis, EMT, and progression of endometriosis.	OTUB1, HSF1	-	In vitro, In vivo	2022	[114]
Sun Y	CHIP activation reduces HMGB1 expression, limiting the energy supply for endometriotic cell growth.	CHIP, HMGB1	YL-109 (CHIP agonist)	In vitro, In vivo, Human sample	2022	[115]
Gou Y	Glycolysis-driven lactate accumulation promotes M2 macrophage polarization, enhancing endometriotic lesion invasion via the Mettl3/Trib1/ERK-STAT3 pathway.	Mettl3, Trib1, ERK, STAT3	2-Deoxy-D-glucose (2-DG)	In vitro, In vivo, Human sample	2023	[116]
Chen Q	Glycolysis-related genes influence immune cell infiltration, contributing to endometriosis progression.	CHPF, CITED2, GPC3, PDK3, ADH6	-	Human sample, In silico	2023	[31]
Wang M	PIM2 enhances glycolysis and fibrosis in endometriotic cells by upregulating PKM2, promoting endometriosis progression.	PIM2, PKM2, HK2, SMH, Desmin, α-SMA	SMI-4a (PIM2 inhibitor)	In vitro, In vivo, Human sample	2023	[108]
Ling X	PFKFB3 enhances glycolysis, proliferation, migration, and invasion of endometriosis cells by stabilizing β-catenin, promoting epithelial-mesenchymal transition (EMT).	PFKFB3, β-catenin	PFK-015 (PFKFB3 inhibitor)	In vitro, In vivo, Human sample	2024	[117]
Sun Y	URKA promotes proliferation, migration, invasion, and glycolysis in ovarian endometriosis by upregulating ERβ.	AURKA, ERβ	Alisertib (AURKA inhibitor)	In vitro, In vivo, Human sample	2024	[118]
Huang ZX	Endometriosis involves dysfunctional CD8+ T cells, where glycolysis and the STAT1/PDCD1 pathway reduce T-cell cytotoxicity and promote lesion growth.	CDK1, CCNB1, STAT1, PDCD1	-	In vitro, In vivo, Human sample, In silico	2024	[119]
Guo S	increased PDK3 and GPC3 levels and decreased ADH6 in EMT patients, which correlated with lower oocyte quality and pregnancy rates.	PDK3, GPC3, ADH6	-	Human sample	2024	[32]
Toniyan KA	endometriomas and pelvic peritoneum lesions, oxygen absorption is significantly reduced, and there is a shift towards succinate utilization, suggesting a Warburg effect with increased glycolysis.	HK2, PK	-	Human sample	2024	[33]
Khashchenko EP	glycolysis reprogramming, mitochondrial biogenesis, and apoptosis suppression drive peritoneal endometriosis	MCT2, PDK1, GLUT1, OPA1, DRP1, Beclin1, Bnip3, ERβ	-	Human sample	2024	[34]
Wen X	H19 expression promotes abnormal glucose metabolism and histone lactylation, driving endometriosis progression.	H19	-	In vitro, In vivo, Human sample	2024	[86]
Gao X	Macrophage-induced ITGB3 upregulation promotes glycolysis and enhances proliferation, migration, and invasion in endometriosis.	ITGB3	-	In vitro, In vivo, In silico	2024	[120]
Sarsenova M	Metabolic reprogramming, including glutathione metabolism, oxidative phosphorylation, and glycolysis, occurs in response to AMPK signaling and HIF-1 signaling perivascular cells of endometriotic lesions.	AMPK, HIF1	-	Human sample, In silico	2024	[30]
Lu J	PAK5 is upregulated in endometriosis, promoting glycolysis by stabilizing PKM2.	PAK5, PKM2	GNE 2861 (PAK5 inhibitor)	In vitro, In vivo, Human sample	2024	[110]
Li J	PKM2/HIF-1α axis regulates glycolysis and plays a key role in endometriosis pathogenesis.	TGFB1, HIF1A, PKM2	-	In vitro, Human sample	2024	[46]
Sarsenova M	Hypoxia-induced TGFBI is involved in fibrosis and angiogenesis and is more highly expressed in ectopic endometrial tissue during the secretory phase.	TGFB1	-	In vitro, Human sample	2024	[47]
Dai F	PTEN deficiency in ectopic endometrial stromal cells (EESCs) promotes glycolytic activity, which enhances M2 macrophage polarization and increases CCL2 production.	PTEN, CCL2	-	In vitro	2025	[62]

**Table 2:** Comparative analysis of the candidate therapeutic agents targeting “glycolysis” and “endometriosis”: mechanisms, development stages, efficacy, and safety.

Drug Name	Mechanism	Development Stage	Efficacy	Safety
Gui-Zhi-Fu-Ling Capsules	Promotes blood circulation and removes blood stasis	Approved for clinical use (in China)	Anti-inflammatory effects, alleviates abdominal pain, regulates menstruation	Generally safe; lacks standardized clinical data
Dichloroacetate (DCA)	Inhibits pyruvate dehydrogenase kinase (PDK) to normalize glucose metabolism	Preclinical/Phase 2	Reduces peritoneal lactate levels and lesion size (animal models)	Potential neurotoxicity with prolonged use
Atorvastatin + Resveratrol	Inhibits GLUT-1/3 and MCT-1/4 to suppress glycolysis and angiogenesis	Preclinical	40% reduction in lesion size and angiogenesis density	Muscle toxicity risk (statin-related)
*Caesalpinia sappan* Extract	PDK1 inhibition induces mitochondrial ROS and apoptosis	Preclinical	>50% apoptosis in 12Z cells	Limited human toxicity data
KRIBB11	HSF1 inhibition reduces PFKFB3 and glycolysis	Preclinical	60% lesion weight reduction in animal models	Potential heat shock protein dysregulation
shLDHA	LDHA gene silencing to inhibit lactate production	Early research stage	70% inhibition of 12Z cell migration	Unconfirmed long-term safety of gene therapy
Cinnamic Acid	Suppresses NF-κB/PKM2 signaling to reduce invasiveness	Preclinical	55% reduction in stromal cell invasion	Mucosal irritation at high doses
*Prunella vulgaris* Extract	LDHA/PDK1 inhibition disrupts mitochondrial function	Preclinical	40% decrease in oxygen consumption rate in 12Z cells	Traditional use history but lacking modern toxicity data
YL-109	CHIP activation promotes HMGB1 ubiquitination and degradation	Preclinical	65% lesion size reduction in animal models	Unclear immune system impacts
2-Deoxy-D-glucose (2-DG)	Glycolysis blockade via glucose analog	Preclinical	75% disease progression inhibition in mice	Hypoglycemia risk
SMI-4a	PIM2 inhibition reduces PKM2 expression and fibrosis	Preclinical	50% reduction in lesion fibrosis in animal models	Potential hematological side effects
PFK-015	PFKFB3 inhibition destabilizes β-catenin	Preclinical	60% inhibition of endometrial cell migration	Risk of erythrocyte dysfunction
Alisertib	AURKA inhibition suppresses ERβ expression and glycolysis	Preclinical	40% reduction in peritoneal lactate levels in mice	Potential mitotic disruption
GNE 2861	PAK5 inhibition blocks PKM2 phosphorylation	Preclinical	70% lesion size reduction in PAK5-KO mice	Unconfirmed neurological effects

## Data Availability

No new data were created or analyzed in this study. Data sharing is not applicable.
